# Determinants of coexistence of undernutrition and anemia among children aged 6–59 months in Nepal: Evidence from the 2022 Nepal Demographic and Health Survey

**DOI:** 10.1371/journal.pone.0339985

**Published:** 2026-01-28

**Authors:** Bikram Adhikari, Biraj Neupane, Jessica Rice, Niharika Jha, Kajol Dahal, Parash Mani Sapkota, Archana Shrestha, Yu Jiang, Xinhua Yu

**Affiliations:** 1 School of Public Health, University of Memphis, Memphis, Tennessee, United States of America; 2 Sindhu Research and Implementation Institute, Sunkoshi, Sindhupalchok, Nepal; 3 Informatics Program, School of Information Science, University of Illinois Urbana-Champaign, Urbana-Champaign, Illinois, United States of America; 4 East Tennessee State University, Johnson, Tennessee, United States of America; 5 HERD International, Karyabinayak, Nepal; 6 School of Public Health, Kathmandu University School of Medical Sciences, Dhulikhel, Nepal; 7 Institute of Implementation Science and Health, Kathmandu, Nepal; Lusofona University of Humanities and Technologies: Universidade Lusofona de Humanidades e Tecnologias, PORTUGAL

## Abstract

**Introduction:**

Undernutrition and anemia among children aged 6–59 months are significant public health issues in developing countries like Nepal. The coexistence of these conditions impacts childhood development. This study aimed to determine the prevalence of undernutrition and anemia, assess their coexistence, and identify contributing factors among children aged 6–59 months in Nepal.

**Methods:**

We conducted a secondary analysis using data from 2022 Nepal Demographic and Health Survey (NDHS 2022), a nationally representative cross-sectional survey. The outcome variables were undernutrition, anemia, and their coexistence. Undernutrition was defined as the presence of stunting, wasting, underweight, or any combination of these conditions. Anemia was defined as hemoglobin levels <11.0 g/dL (adjusted for altitude). We applied multivariable multinomial logistic regression to determine factors associated with coexistence, and multivariable logistic regression to assess factors associated with undernutrition and anemia separately. We presented the results from the regression analysis using adjusted odds ratio (aOR) and 95% confidence intervals (CI).

**Results:**

Among 2,395 children, the weighted prevalence of undernutrition was 33.5%, anemia was 43.4%, and coexistence of undernutrition and anemia was 16.0%. Children from the richest wealth quintile, whose mothers had at least secondary education, and those whose mothers participated in household decision-making had 53% (aOR: 0.47; 95% CI: 0.26 to 0.86), 48% (aOR:0.52; 95% CI: 0.32 to 0.86), and 34% (aOR: 0.66; 95% CI: 0.47 to 0.94) lower odds of experiencing the coexistence of undernutrition and anemia compared to their counterparts. Children with underweight mothers had 80% (aOR:1.80; 95% CI:1.20 to 2.70) higher odds of coexistence compared to their counterparts.

**Conclusion:**

The prevalence of undernutrition, anemia, and their coexistence among children was high in Nepal. Interventions that improve mothers’ education, strengthen their roles in the family, and enhance the household financial condition are essential to reduce these conditions and improve children’s nutritional status.

## Introduction

Undernutrition, including stunting, wasting, and underweight, is a major public health challenge among children under 5 years (U-5) of age globally. This issue is particularly pronounced in developing countries like Nepal [1–4]. There are three different causes of undernutrition – immediate, underlying, and basic. The immediate causes include inadequate diet intake and repeated illnesses; the underlying causes include suboptimal child feeding and caregiver practices from mothers, lack of access to healthcare facilities, poverty, and food insecurity; basic causes include limited information, political and economic insecurity, gender inequality, and the occurrence of natural disasters [5].

Different indices of undernutrition reflect different underlying development status. Stunting indicates chronic malnutrition reflecting long-term nutrient deficiencies and is linked with delayed and impaired motor and cognitive development [6]. Wasting indicates acute malnutrition associated with recent severe food shortages or illnesses leading to weight loss and is a strong predictor of mortality [6]. Underweight combines information about linear growth obstruction and weight for length or height [7].

Anemia, particularly iron deficiency anemia, is another common and pressing public health concern among children U-5 in low-, middle- and high-income countries. It results from poor nutrition and has severe adverse health consequences including impaired cognitive development, impaired immunity, disability, and an increased risk of morbidity and mortality [8–10].

In 2022, the global prevalence of stunting among children under five years of age was 22.3%, while in South Asia, it reached 31.8%. Additionally, the global prevalence of wasting was at 6.8%, with South Asia reporting a prevalence of 14.8% [11]. In 2019, global anemia prevalence was 39.8% [12]. In Nepal, the prevalence of stunting, and wasting among U-5 children was 25% and 8% respectively, and the prevalence of anemia was 43% among children 6–59 months old [13].

The coexistence of undernutrition and anemia increases the risk of childhood morbidity and mortality [14–16]. Almost fifty percent of deaths among children under the age of five are associated with undernutrition, which predominantly occurs in low- and middle-income countries (LMICs). Notably, 88% of these countries (124 out of 141) experience multiple forms of malnutrition [17]. The developmental, economic, social, and medical impacts of undernutrition and anemia among children are serious and perpetuating, for individuals and their families, communities, and countries [7].

Furthermore, anemia and undernutrition are both concentrated in socioeconomically disadvantaged groups, which share numerous multifaceted causes and complex interactions between diet, transmissible illnesses, inadequate care and unhealthy household environments that adversely affect the cognitive development and physical well-being of children [18–20]. For instance, lower mother’s education level and socioeconomic status of households are linked with higher rates of malnutrition among children [18].

Though the concerns on nutrition should be explored beyond any single form of malnutrition to the coexistence of multiple forms, most studies in Nepal have focused on undernutrition and anemia separately. A few studies that have examined both conditions together primarily reported that children from poorer households, mothers with low education levels, and those with inadequate dietary diversity were more likely to experience concurrent undernutrition and anemia [21,22]. Even when both malnutrition and anemia were considered, anemia was often treated merely as a correlating factor or predictor rather than as part of a combined outcome reflecting their simultaneous occurrences. Moreover, there remains a gap in understanding how maternal factors (such as nutritional status and exposure to health information) and household-level socioeconomic characteristics interact to influence the coexistence of undernutrition and anemia among children under five in Nepal.

Therefore, this study aims to ascertain the prevalence of stunting, wasting, and underweight, as well as their coexistence, and the coexistence of undernutrition and anemia in Nepal. It also assesses the association of undernutrition, anemia, and coexistence of undernutrition with household economic status, maternal education and nutritional status, and exposure to televised or broadcasted health programs among children aged 6–59 months in Nepal. By identifying these factors, this study helps inform the development of targeted interventions and policies designed to mitigate malnutrition within this vulnerable population, thereby enhancing child health outcomes and alleviating the burden of malnutrition in Nepal.

## Methods

### Data source

In this study, we analyzed data from the Nepal Demographic and Health Survey (NDHS) conducted in 2022 [23]. NDHS 2022 is the nationally representative survey implemented by New ERA under the aegis of the Ministry of Health and Population (MoHP) with the technical support of ICF International and funding from the United States Agency for International Development (USAID) [13]. The NDHS follows standardized demographic and health survey (DHS) protocols for sampling, data collection, and quality assurance.

### Ethical approval

We received permission from the official website of “the DHS program” (https://www.dhsprogram.com) to download and use (*submitted on: 07/25/2023 and approved on 07/26/2023*) NDHS 2022 dataset [23]. NDHS 2022 obtained ethical approval from the Institutional Review Board of ICF International, United States of America (*Reference number: 180657.0.001.NP.DHS.01, Date: 28 April 2022)* and the ethical review board of Nepal Health Research Council (*Reference number: 678, Date: 30 September 2021*) [13]. In the NDHS 2022, informed consent was taken from the participants before enrolling them into the study.

### Study setting

This study used nationally representative data from Nepal, a landlocked country located in Southeast Asia with an area of 147,516 km^2^ [24]. It has seven administrative provinces, within which lies 753 municipalities (6 metropolitan cities, 11 sub-metropolitan cities, 276 urban municipalities, 460 rural municipalities) [24]. Nepal has three ecological belts- Mountain, Hill, and Terai. Based on the 2021 Census, the total population of Nepal was 29,164,578 of which 14,911,027 (51.1%) were females and 14,253,551 (48.9%) were males [24]. Based on the Nepal Human Development Report 2020, Nepal was ranked 147^th^ based on the human development index (HDI) with an overall HDI of 0.587 (rural: 0.647; urban: 0.561) [25]. According to the Global Hunger Index, Nepal has a ranking of 19.5, implying moderate food insecurity and undernourishment risk [26].

### Sampling and data collection in NDHS

The NDHS uses a two-stage stratified cluster sampling design. In the first stage, wards were selected as primary sampling units using probability proportional to size. In the second stage, households within each cluster were selected through systematic random sampling. Selected households were contacted through in-person visits, during which trained field enumerators listed all usual residents and visitors. All women aged 15–49 years who were either permanent residents or stayed in the selected household the night before the survey were invited to participate. In a sub-sample of households, men aged 15–49 years were also invited. In total, the 2022 NDHS interviewed 13,786 households out of 13833 occupied household (response rate: 99.7%). This yielded completed interviews from 14,845 women, 4,913 men, and proxy-reported information for 2,643 children under five [13]. Data collection was done in NDHS using standardized questionnaires (household, women’s, men’s, and biomarker questionnaires), administered through face-to-face interviews using tablet-based data entry. In this study, we analyzed the data of 2,395 children aged 6–59 months using the NDHS 2022 dataset.

### Measures

#### Outcome variables.

The primary outcome was “Coexistence of undernutrition (stunting, wasting or underweight) and anemia”. Anemia was assessed using altitude-adjusted hemoglobin levels and a child was considered anemic if the level was less than 11.0 g/dL [13]. A child was classified to have undernutrition if they exhibit either stunting, wasting, underweight, or any of these conditions, as based on the composite index of anthropometric failure (CIAF) concept [27]. In the NDHS survey, weight was measured using SECA scales (model SECA874U). Height and length were measured with a measuring board (Shorrboard). Anemia was measured with blood specimens via finger-prick or heel-prick (for children 6–12 months of age). Hemoglobin was analyzed using a portable HemoCue device [13].

Children whose height-for-age z-score was below minus two standard deviations (–2 SD) from the median of the WHO child growth standards were defined as stunted [28]. Children whose weight-for-height z-score was below –2 SD from the median of the WHO child growth standards were defined as wasted [28]. Children whose weight-for-age z-score was below –2 SD from the median of the WHO child growth standards were defined as underweight [28].

The outcome variable coexistence of undernutrition and anemia consists of four categories a) Normal (having neither undernutrition nor anemia), b) only undernutrition (having only undernutrition), c) only anemia (having only anemia), and d) coexistence of undernutrition-anemia (having both undernutrition and anemia).

#### Exposure variables.

The exposure variables for this study were wealth quintile (poorest/poorer/middle/richer/richest), maternal body mass index (underweight/normal/overweight or obese), mother’s education (no education/ basic level education/Secondary and higher level), mother’s exposure to health programs on television and radio (yes/no), and mother’s participation in household decision-making (yes/no). The definition of each exposure variable is explained in Table 1 below*:*

**Table 1:** List of exposure variables with their definition.

Variables	Definition
Age of child	The age of the child was collected in months. We recategorized it into 6–12 months (late infant), 13–36 months (toddler) and 37–59 months (pre-school).
Wealth quintile	The wealth quintile measures the economic status of the household, and it is classified into poorest, poorer, middle, richer, and richest based on the wealth index. The wealth index included in the NDHS is a composite measure constructed using principal component analysis based on household assets, housing materials, water and sanitation facilities, and ownership of durable goods; households were assigned to five wealth quintiles (poorest to richest).
Mother’s education	Mother’s education was classified into basic education (grades 1–8), secondary level education (grades 9–12), or higher education (grades 13 and above) based on the DHS questionnaire.
Mother’s exposure to Health program in TV/radio	The women were considered to have exposure to specific health programs if they heard or saw health programs on the radio or television (TV). This is measured based on the DHS questionnaire. These programs included eight different health-related programs broadcasted on radio and TV namely *Jana Swasthya Bahas* (Public Health Debate) television program, *Jeevan Chakra* (Life Cycle) television serial, *Jana Swasthya* (Public Health) radio program, *Swasthya Gatibidhi* (Health Affairs) radio program, *Eak Dui Tin Sunau eekai Chhin* (Listen for a While) radio program, *Bhanchhin Ama* (Mother Says) radio program, *Hello Bhanchin Ama* (Mother Says Hello) radio program and *Jeewan Rakshya* (Save Life COVID- response) radio programs [29].
Mother’s participation in household decision-making	A composite variable measured from the mother’s participation (alone or with her husband) in making three household decisions (access to healthcare, major household purchases, and visits her family or relatives) grouped into no participation or participation in decision-making [30,31].
Mother’s body mass index (BMI)	The mother’s BMI was classified as underweight, normal, overweight, and obese. The mother was considered underweight if BMI is less than 18.5 kg/m^2^, normal if BMI is between 18.5 to 24.9 kg/m^2^, overweight if her BMI is between 25 to 29.9 kg/m^2^, and obese if her BMI is greater than 30.0 kg/m^2^ [32].

#### Potential confounding variables.

Based on the existing literature, some variables were found to be potential confounders in the association between outcome and exposure. Confounding variables included sociodemographic variables consisting of place of residence (rural/urban), and ecological belt (mountain/hill/terai), child-related variable consisting of sex of the child (male/female), and parents-related variables consisting of parity (Primipara/Multipara), mother’s anemia status (a mother was considered anemic if the level was less than 11.0 g/dL. [13]) and father’s education (no education/basic level/secondary and higher level).

### Statistical analysis

We conducted pre-analytical processing and statistical analysis using R version 4.3.2 [33] and R studio [34]. We carried out weighted descriptive and inferential analyses using the “survey” package to address complex survey design and non-response. Parametric numerical variables were presented as mean (standard deviation) and non-parametric numerical variables as median (interquartile range). We presented categorical variables as frequency, percent, and their 95% confidence interval (CI). We computed the prevalence and 95% CI using the Wilson method.

We performed multivariable multinomial logistic regression to determine the association between the coexistence of undernutrition and anemia (none/undernutrition only/anemia only/coexistence) and the exposure variables listed in Table 1, adjusting for potential confounding variables (place of residence, ecological belt, age of the child, sex of the child, mother’s parity, father’s education). We performed binary logistic regression to determine the association between undernutrition or anemia with the exposure variables. We checked for multicollinearity using variance inflation factor (VIF) and removed province (VIF = 13.31, greater than the cutoff of 10) from the regression models, as the information provided by the province was contained by the region and urban status variables. All regression analyses were conducted using complete cases after excluding observations with missing data. We calculated and presented crude odds ratio (cOR) and adjusted odds ratio (aOR) and their 95% CI.

## Results

Table 2 presents the sociodemographic characteristics of 6–59 months of children stratified by age. Of the total U-5 children, 48.5% were female. Age distribution showed 10.4% were 6–12 months old, 45.8% were 13–36 months old, and 43.8% were 37–59 months old. Most children (59.1%) were from the Terai region, while the fewest (5.6%) were from the mountain region. The ratio of children from urban to rural areas was 2:1. By province, the majority were from Madhesh (26.4%), followed by Koshi (17.9%), Lumbini (16.7%), and Bagmati (15.4%).

**Table 2:** Characteristics of 6–59 months children.

Characteristics	Overall, %	6–12 months children, %	13–36 months children, %	37–59 months children, %
*Total unweighted frequency, n*	2395	256	1092	1047
*Total weighted frequency, n*	2335	244	1069	1022
**Age of children in months,** %		10.4	45.8	43.8
**Sex, %**				
Male	51.5	52.6	51.4	51.3
Female	48.5	47.4	48.6	48.7
**Ecological region, %**				
Mountain	5.6	5.6	5.6	5.6
Hill	35.3	41.7	34.4	34.7
Terai	59.1	52.7	60	59.8
**Type of place of residence, %**				
Urban	64.3	63.1	64.2	64.7
Rural	35.7	36.9	35.8	35.3
**Province, %**				
Koshi	17.9	18.7	17.2	18.5
Madhesh	26.4	20.7	27.2	27
Bagmati	15.4	20	15.8	13.9
Gandaki	7.2	6.7	6.1	8.5
Lumbini	16.7	15.6	17.1	16.6
Karnali	7.7	9.8	7.1	7.8
Sudurpashchim	8.6	8.5	9.6	7.7
**Wealth quintile, %**				
Poorest	24.5	22.2	24.4	25
Poorer	22	19.9	23.5	20.9
Middle	20.7	19.6	19.6	22
Richer	18.1	19.1	19.2	16.8
Richest	14.7	19.2	13.3	15.2
**Mother’s education, %**				
No education	22.2	15.3	23.1	22.9
Basic	35.9	32.5	35.7	37
Secondary and higher	41.9	52.2	41.2	40
*Missing, n*	55	0	14	40
**Father’s education, %**				
No education	10.7	6.1	10.8	11.8
Basic	41	42.2	41.1	40.7
Secondary and higher	48.3	51.7	48.1	47.6
*Missing, n*	322	27	135	160
**Mother’s age at delivery, %**				
< 20 years	19.8	18.3	19.2	20.8
20–34 years	76.3	78.9	77.7	74.2
35–49 years	3.9	2.8	3.1	5
*Missing, n*	259	21	104	134
**Parity, %**				
Primipara	32.1	41.3	37.3	24.2
Multipara	67.9	58.7	62.7	75.8
*Missing, n*	259	21	104	134
**Mother’s BMI, %**				
Underweight (<18.5 kg/m^2^)	14.6	12.2	17.8	11.9
Normal (18.5 to 24.9 kg/m^2^)	61.7	58.1	62.2	62.1
Overweight or obese (>=25 kg/m^2^)	23.7	29.7	20	26
*Missing, n*	399	22	185	192
**Mother’s participation in household decision-making, %**				
No participation	33.5	32.5	34.6	32.6
Participation	66.5	67.5	65.4	67.4

*n: weighted frequency; %: weighted percentage*

Missing are presented as numbers

All percentages are column percentage.

**Fig 1:**
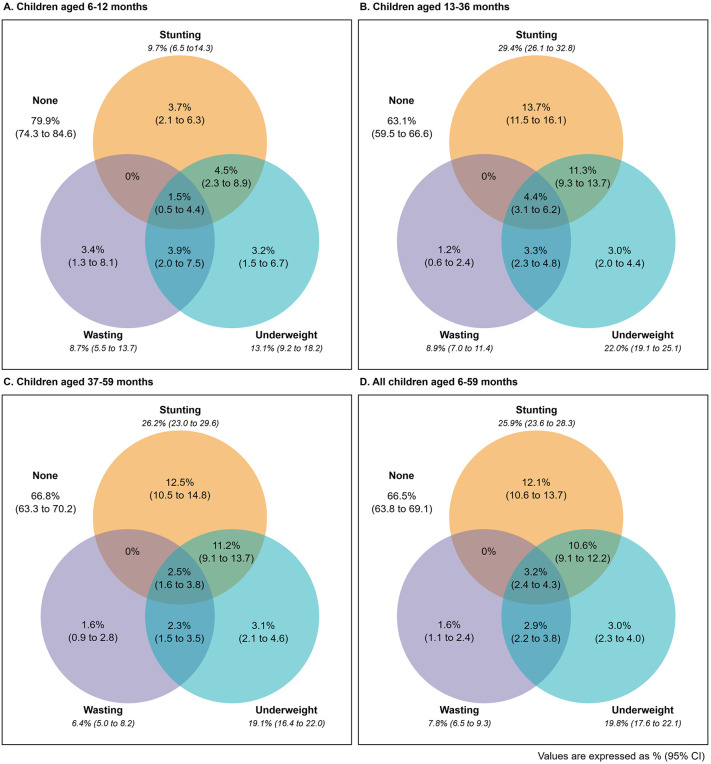
Prevalence of coexistence of different forms of undernutrition among A) children aged 6–12 months, B) children aged 13–36 months, C) children aged 37–59 months and D) all children aged 6–59 months.

Fig 1 presents the prevalence of stunting, wasting and underweight among Nepalese children aged 6–59 months. The prevalence of stunting, wasting, and underweight were 25.9% (95% CI: 23.6 to 28.3), 7.8% (95% CI: 6.5 to 9.3), and 19.8% (95% CI: 17.6 to 22.1), respectively. Of all children, 3.2% (95% CI: 2.5 to 4.3) had all three conditions, 13.5% (95% CI: 11.9 to 15.3) had two of the three conditions, and 16.8% (95% CI: 15.2 to 18.5) had one of the three conditions.

**Fig 2:**
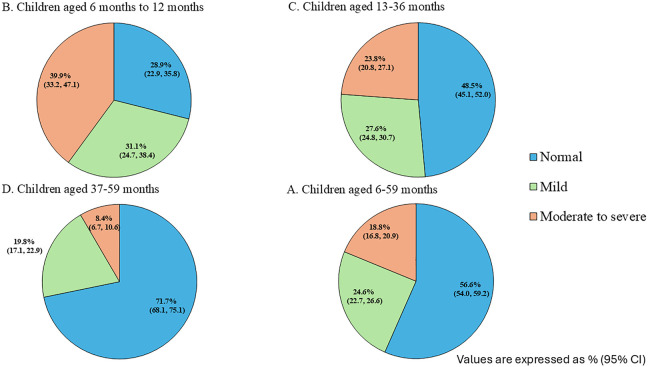
Prevalence of anemia among A) children aged 6–12 months, B) children aged 13–36 months, C) children aged 37–59 months and D) all children aged 6–59 months.

The prevalence of anemia was 43.4% (95% CI: 40.9 to 45.9), which included 18.8% (95% CI:16.8 to 20.9) with moderate to severe anemia and 24.6% (95%CI: 22.7 to 26.6) with mild anemia. The prevalence of moderate to severe anemia was highest in 6–12 months children, accounting for 39.9% (95% CI: 33.2 to 47.1) and decreased to 8.4% (95% CI: 6.7 to 10.8) among 37–59 months children (Fig 2).

**Fig 3:**
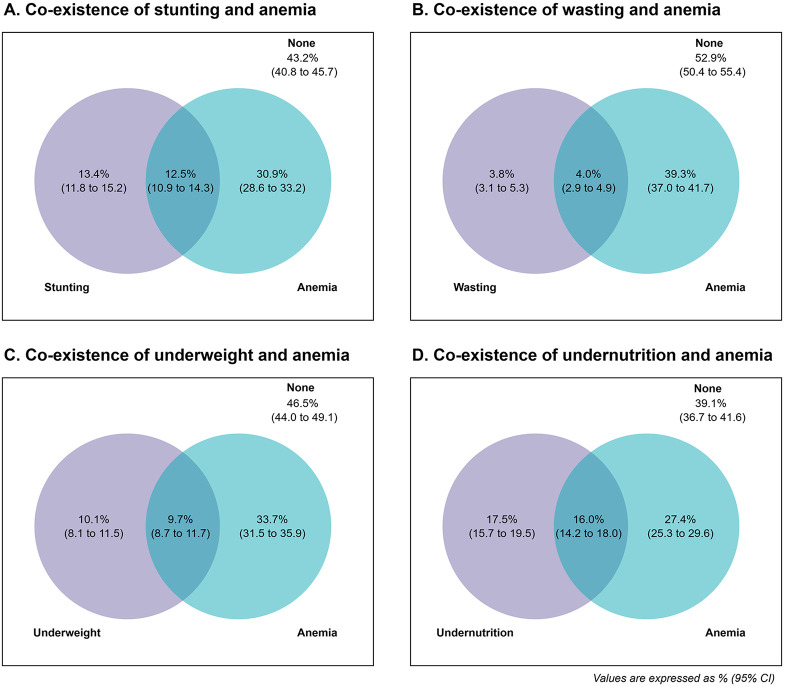
Prevalence of coexistence of anemia with stunting, wasting, underweight, and overall undernutrition.

The prevalence of coexistence of stunting-anemia, wasting-anemia, underweight-anemia, undernutrition-anemia were 12.5% (95%CI: 10.9 to 14.3), 4.0% (95%CI: 2.9 to 4.9), 9.7% (95% CI: 8.7 to 11.7), and 16.0% (95% CI: 14.2 to18.0) respectively (Fig 3A-D). Of all children, 1.9% (95%CI: 1.3 to 2.6) had all three undernutrition conditions and anemia, 6.4% (95%CI: 5.3 to 7.8) had two undernutrition conditions and anemia, and 7.6% (95%CI: 6.6 to 8.9) had one undernutrition condition and anemia. (***not presented in tables***)

Table 3 presents the prevalence of coexistence of anemia and undernutrition by age group and sex of child. The prevalence of coexistence of undernutrition and anemia was highest in children 13–36 months (overall: 22.0%, male: 20.4%, female: 23.7%) followed by 6–12 months old children (overall: 16.2%, male: 17.4%, female: 14.9%). Prevalence of anemia only was highest in 6–12 months (overall: 54.9%, male: 50.2%, female: 60.1%) and only undernutrition in 37–59 months old children (overall: 23.5% male: 21.0%, female: 26.2%).

**Table 3:** Coexistence of anemia and undernutrition conditions (n = 2,335).

Characteristic	6–59 months children, % (95%CI)	6–12 months children, % (95%CI)	13–36 months children, % (95%CI)	37–59 months children, % (95%CI)
**All children**				
None	39.1 (36.6 to 41.7)	25.0 (19.4 to 31.6)	33.6 (30.5 to 36.9)	48.2 (44.3 to 52.1)
Only Anemia	27.4 (25.3 to 29.6)	54.9 (48.3 to 61.3)	29.5 (26.4 to 32.7)	18.6 (15.8 to 21.8)
Only undernutrition	17.5 (15.6 to 19.6)	3.9 (1.99 to 7.49)	14.9 (12.5 to 17.6)	23.5 (20.6 to 26.8)
Coexistence of undernutrition and anemia	16.0 (14.1 to 18.0)	16.2 (11.7 to 22.0)	22.0 (19.1 to 25.3)	9.6 (7.89 to 11.7)
**Male children**				
None	39.5 (36.0 to 43.1)	29.5 (21.0 to 39.6)	33.5 (29.0 to 38.4)	48.2 (43.0 to 53.5)
Only Anemia	27.9 (25.1 to 30.9)	50.2 (41.1 to 59.2)	30.7 (26.6 to 35.1)	19.6 (15.9 to 23.9)
Only undernutrition	16.5 (14.2 to 19.2)	2.9 (0.94 to 8.83)	15.4 (12.3 to 19.2)	21.0 (17.2 to 25.5)
Coexistence of undernutrition and anemia	16.1 (13.7 to 18.8)	17.4 (11.3 to 25.9)	20.4 (16.9 to 24.5)	11.2 (8.71 to 14.2)
**Female children**				
None	38.7 (35.4 to 42.1)	20.1 (13.3 to 29.3)	33.8 (29.5 to 38.4)	48.1 (43.0 to 53.3)
Only Anemia	26.8 (23.8 to 30.1)	60.1 (49.2 to 70.0)	28.2 (23.9 to 33.1)	17.7 (13.8 to 22.3)
Only undernutrition	18.6 (15.9 to 21.5)	4.9 (2.09 to 11.2)	14.3 (11.2 to 18.1)	26.2 (21.7 to 31.2)
Coexistence of undernutrition and anemia	15.9 (13.4 to 18.7)	14.9 (9.01 to 23.5)	23.7(19.3 to 28.7)	8.0 (5.72 to 11.1)

*%: weighted percent; CI: confidence interval; All percentages in the table are row percentage*

**Fig 4:**
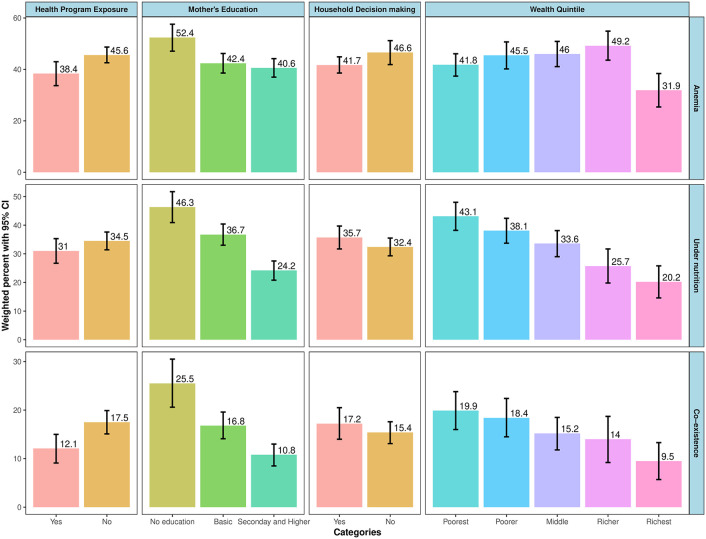
Distribution of anemia, overall undernutrition and their coexistence of anemia across exposure variables (vertical bars represent 95% CIs).

Fig 4 and Table 4 present the distribution of anemia, undernutrition and coexistence across categories of quintile wealth, mother’s education, household decision-making and mother’s exposure to health programs on TV and radio. The prevalence of undernutrition and coexistence was highest in poorest wealth quintile and lowest in richest wealth quintile, and highest in children whose mother had no education.

**Table 4:** Distribution of undernutrition, anemia and their coexistence across exposure variables.

Characteristics	6–12 months children*			13–36 months children*			37–59 months children*		
	Anemia only, %	Undernutrition only, %	Coexistence, %	Anemia only, %	Undernutrition only, %	Coexistence, %	Anemia only, %	Undernutrition only, %	Coexistence, %
**Wealth quintile**									
Poorest	55.9	4.3	19.0	24.2	19.7	26.6	12.3	30.9	13.2
Poorer	51.7	1.8	15.2	30.3	16.4	25.4	17.6	27.4	11.0
Middle	60.9	3.3	12.2	31.0	12.3	21.3	24.4	27.3	10.1
Richer	56.9	4.0	24.0	35.2	12.5	18.5	29.5	13.0	5.9
Richest	48.8	6.2	10.4	27.2	10.4	13.7	10.2	12.4	5.3
**Mother’s education**									
No education	51.7	8.1	20.9	27.5	15.1	35.5	21.9	29.0	15.6
Basic	51.1	1.1	22.4	27.7	16.1	22.9	17.7	27.9	9.4
Secondary and higher	58.1	4.4	11.0	32.3	13.8	13.9	18.0	15.9	7.2
**Participation in household decision-making**	52.9	4.7	14.9	28.5	14.9	21.3	17.9	22.2	9.4
**Exposure to health programs on TV/radio**	64.3	4.7	11.9	24.6	15.5	14.2	16.6	26.7	10.0
**Mother’s BMI**									
Underweight	50.3	6.9	25.3	22.1	22.5	33.4	28.3	22.5	13.2
Normal	58.0	2.9	17.1	30.3	14.4	21.1	15.7	26.2	10.8
Overweight or obese	46.1	5.0	13.9	37.7	10.2	12.9	22.1	18.8	5.5

*%: weighted percentage*

* Row percentage

Table 5 presents the factors associated with undernutrition and anemia among children aged 6–59 months. In univariable regression, undernutrition was significantly associated with wealth quintile, mother’s education, age of child, and maternal nutritional status. In multivariable regression, undernutrition was found to be associated with age of child, wealth quintile, mother’s education, and maternal nutrition status after adjusting for confounding variables. The odds of having undernutrition were 1.96 times among 13–36 months children and 1.70 times in the 37–59 months children compared to 6–12 months children. The odds of undernutrition were 44% (aOR: 0.56, 95%CI: 0.34 to 0.92) lower in children from the richer wealth quintile compared to the poorest, 36% (aOR: 0.64, 95%CI: 0.45 to 0.93) lower in the child whose mother had secondary or higher-level education. We did not find a significant association between undernutrition and mother participation in household decision-making and exposure to health programs on TV/radio.

**Table 5:** Factors associated with undernutrition among children aged 6–59 months.

Characteristic	Presence of undernutrition, n (%) *	Unadjusted models			Adjusted model^#^		
		cOR	95% CI	p-value	aOR	95% CI	p-value
**Age of child**							
6–12 months	49 (20.1)	Ref			Ref		
13–36 months	394 (36.9)	2.32	1.67 to 3.22	<0.001	1.96	1.33 to 2.90	<0.001
37–59 months	339 (33.2)	1.97	1.39 to 2.81	<0.001	1.70	1.12 to 2.59	0.013
**Wealth quintile**							
Poorest	246 (43.1)	Ref			Ref		
Poorer	195 (38.1)	0.81	0.62 to 1.06	0.122	0.93	0.67 to 1.30	0.680
Middle	162 (33.6)	0.67	0.50 to 0.88	0.005	0.78	0.54 to 1.13	0.194
Richer	109 (25.7)	0.46	0.32 to 0.66	<0.001	0.53	0.34 to 0.82	0.005
Richest	70 (20.2)	0.33	0.22 to 0.50	<0.001	0.56	0.34 to 0.92	0.024
**Mother’s education**							
No education	234 (46.3)	Ref			Ref		
Basic	301 (36.7)	0.67	0.52 to 0.88	0.003	0.87	0.65 to 1.16	0.332
Secondary and higher	231 (24.2)	0.37	0.28 to 0.49	<0.001	0.64	0.45 to 0.93	0.019
**Mother’s Participation in household decision making**							
No participation	279 (35.7)	Ref			Ref		
Participation	503 (32.4)	0.86	0.70 to 1.07	0.173	0.82	0.63 to 1.06	0.133
**Exposure to health programs on TV/radio**							
No	615 (34.5)	Ref			Ref		
Yes	145 (31.0)	0.85	0.67 to 1.09	0.199	0.92	0.69 to 1.24	0.595
**Mother’s BMI**							
Normal	411 (34.5)						
Underweight	132 (46.6)	1.66	1.23 to 2.23	<0.001	1.48	1.09 to 2.01	0.013
Overweight or obese	106 (23.1)	0.57	0.43 to 0.75	<0.001	0.75	0.56 to 1.01	0.057

n: weighted frequency; %: weighted percent; Ref: reference group; cOR: crude odds ratio; aOR: adjusted odds ratio; CI: confidence interval; * Row percent

*# adjusted for sex of child, father’s education, parity, place of residence, ecological belt, mother’s age at childbirth, and adjusted for each other*

Table 6 presents the factors associated with anemia among children aged 6–59 months. The factors associated were age of the child, wealth quintile, and anemia status of the mother. The odds of having anemia was 41% (aOR: 0.59, 95%CI: 0.36 to 0.97) lower among children belonging to richest quintile compared to poorest. The odds of having anemia was 64% (aOR: 0.36, 95%CI: 0.24 to 0.53) lower among children aged 13–36 months and 87% (aOR: 0.13; 95%CI: 0.09 to 0.20) lower child aged 37–59 months compared to child aged 6–12 months after adjusting for potential confounding variables.

**Table 6:** Factors associated with anemia among children aged 6–59 months.

Characteristic	Prevalence of anemia, n (%) *	Unadjusted models			Adjusted model^#^		
		cOR	95% CI	p-value	aOR	95% CI	p-value
**Age of child**							
6–12 months	173 (71.1)	Ref			Ref		
13–36 months	551 (51.5)	0.43	0.31 to 0.60	<0.001	0.36	0.24 to 0.53	<0.001
37–59 months	289 (28.3)	0.16	0.11 to 0.23	<0.001	0.13	0.09 to 0.20	<0.001
**Wealth quintile**							
Poorest	239 (41.8)	Ref			Ref		
Poorer	233 (45.5)	1.16	0.88 to 1.53	0.291	0.88	0.63 to 1.23	0.451
Middle	222 (46.0)	1.19	0.90 to 1.56	0.215	0.89	0.61 to 1.30	0.531
Richer	209 (49.2)	1.35	1.02 to 1.80	0.038	1.07	0.72 to 1.60	0.725
Richest	110 (31.9)	0.65	0.46 to 0.93	0.02	0.59	0.36 to 0.97	0.037
**Mother’s education**							
No education	265 (52.4)	Ref			Ref		
Basic	347 (42.4)	0.67	0.52 to 0.86	0.002	0.80	0.57 to 1.13	0.210
Secondary and Higher	388 (40.6)	0.62	0.48 to 0.80	<0.001	0.84	0.56 to 1.26	0.409
**Mother’s Participation in decision making**							
No participation	365 (46.6)	Ref			Ref		
Participation	648 (41.7)	0.82	0.65 to 1.03	0.095	0.76	0.55 to 1.05	0.099
**Exposure to health programs on TV/radio**							
No	813 (45.6)	Ref			Ref		
Yes	179 (38.4)	0.74	0.59 to 0.94	0.012	1.00	0.74 to 1.35	0.990
**Mother’s BMI**							
Underweight	149 (52.5)	1.46	1.07 to 1.98	0.016	1.20	0.84 to 1.71	0.319
Normal	516 (43.2)	Ref			Ref		
Overweight or obese	189 (41.1)	0.92	0.70 to 1.21	0.548	1.19	0.87 to 1.63	0.286

*n: weighted frequency; %: weighted percent*

* Row percent

*Ref: reference group; cOR: crude odds ratio; aOR: adjusted odds ratio; CI: confidence interval*

*# adjusted for sex of child, father’s education, parity, place of residence, ecological belt, mother’s age at childbirth, mother’s anemia status, and adjusted for each other*

Table 7 presents the factors associated with coexistence of undernutrition and anemia among children aged 6–59 months. The coexistence of undernutrition and anemia was significantly associated with the richest wealth quintile, the mother’s education status, the mother’s BMI, and presence of anemia in children. The children from the richest wealth quintile were 53% (aOR: 0.47; 95%CI: 0.26 to 0.86) less likely to have coexistence of undernutrition and anemia than children from the poorest wealth quintile. The children whose mothers had secondary or higher-level education had 48% (aOR: 0.52; 95%CI: 0.32 to 0.86) lower odds of having coexistence compared to children whose mothers had no formal education. The odds of having coexistence of undernutrition and anemia was 34% (aOR: 0.66; 95%CI: 0.47 to 0.94) lower in the children whose mother participate in household decision-making after adjusting for confounding variables. The child whose mother was underweight was 1.80 times (95%CI: 1.20 to 2.70) more likely and mother with anemia were 2.43 times more likely to have coexistence compared to a child whose mother had normal nutrition status.

**Table 7:** Factors associated with coexistence of undernutrition and anemia among children aged 6–59 months.

Exposure variables	Anemia only vs Normal			Undernutrition only vs Normal			Coexistence vs Normal		
	aOR^#^	95% CI	p-value	aOR^#^	95% CI	p-value	aOR^#^	95% CI	p-value
**Age of child** *(ref: 6*–*12 months child)*									
13–36 months	0.37	0.25 to 0.54	<0.001	3.02	1.32 to 6.91	0.009	0.68	0.42 to 1.11	0.123
37–59 months	0.16	0.11 to 0.24	<0.001	3.16	1.39 to 7.20	0.006	0.21	0.12 to 0.35	<0.001
**Wealth Quintile** *(ref: Poorest)*									
Poorer	0.94	0.63 to 1.40	0.77	0.98	0.65 to 1.48	0.928	0.8	0.51 to 1.25	0.325
Middle	0.98	0.64 to 1.49	0.917	0.83	0.53 to 1.30	0.412	0.61	0.37 to 1.01	0.054
Richer	1.04	0.67 to 1.61	0.869	0.48	0.28 to 0.81	0.006	0.58	0.34 to 0.99	0.048
Richest	0.49	0.30 to 0.79	0.004	0.38	0.21 to 0.67	0.001	0.47	0.26 to 0.86	0.015
**Mother’s education** *(ref: no education)*									
Basic	0.87	0.60 to 1.27	0.473	1.00	0.67 to 1.49	0.996	0.70	0.46 to 1.06	0.094
Secondary and higher	1.02	0.67 to 1.56	0.923	0.87	0.54 to 1.38	0.543	0.52	0.32 to 0.86	0.010
**Participation in HH decision**-making *(ref: nonparticipation)*	0.69	0.51 to 0.93	0.016	0.73	0.52 to 1.02	0.064	0.66	0.47 to 0.94	0.02
**Exposure to health programs on TV/radio** *(ref: No)*	1.04	0.77 to 1.42	0.783	1.00	0.70 to 1.42	0.996	0.89	0.59 to 1.34	0.572
**Mother’s BMI** *(ref: normal weight)*									
Underweight	1.17	0.80 to 1.71	0.426	1.44	0.96 to 2.16	0.075	1.80	1.20 to 2.70	0.005
Overweight and obese	1.30	0.97 to 1.74	0.076	0.89	0.63 to 1.28	0.539	0.88	0.59 to 1.32	0.529

***Normal*** *participants having neither undernutrition nor anemia*

***ref:*** *reference group****; aOR:*** *adjusted odds ratio****; CI:*** *confidence interval*

***Bold*** *represents significance at 95% confidence level*

^**#**^ *adjusted for sex of child, father’s education, parity, place of residence, ecological belt, mother’s age at childbirth, mother’s anemia status, and adjusted for each other*

## Discussion

Our analysis of a national survey revealed significant issues related to undernutrition, anemia and their coexistence among Nepalese children in 2022. Overall, the prevalence of undernutrition and anemia was 33.5% and 43.4%, respectively. The prevalence of coexistence between undernutrition and anemia was 16.0%. Specifically, the coexistence of stunting, wasting, and underweight with anemia was 12.5%, 4.0%, and 9.7%, respectively. Undernutrition demonstrated significant associations with age of child, household wealth, mother’s education, and mother’s BMI. Anemia was associated with household wealth and age of child. The coexistence of undernutrition and anemia exhibited significant associations with wealth, maternal education, maternal involvement in household decision-making, and mother’s BMI. However, none of the three outcomes (undernutrition, anemia, and their coexistence) were found to be associated with maternal exposure to health programs on television and radio.

A study in Ethiopia reported a higher prevalence of undernutrition among preschool children (50.8%) and among children aged 6–59 months (57.3%) [35]. These figures are higher than the prevalence of undernutrition among 6–59 months children in Nepal (33.5%) found in our study. Our findings indicate that Nepal’s burden, though lower, remains substantial. These differences may reflect variations in economic development, food security, and maternal and child health interventions across countries [36].

The prevalence of anemia among 6–59 months children in our study (43.5%) was higher than the global prevalence (39.8%) but lower than that reported among African children (60.2%) [12]. Our study also demonstrates a 9.1 percentage point reduction in anemia prevalence between 2016 (52.6%) [37] and 2022, indicating progress in addressing this issue. Comparable rates have been observed in neighboring countries. A meta-analysis conducted by Kundu *et al* in Bangladesh reported a prevalence of 46.8% [38], which was similar to our finding. The latest National Family Health Survey (2019−21) in India reported a prevalence of 67.1%, which is higher than that observed in Nepal [39]. The decline in anemia prevalence in Nepal is encouraging but underscores the need for sustained efforts to address this condition, particularly among vulnerable populations [23].

The prevalence of anemia was highest in 6–12 months (39.9%) and lowest in 37–59 months (8.4%) whereas prevalence of undernutrition was lowest in 6–12 months (20.0%) and highest in 13–36 (36.9%) followed by 37–59 months children (33.2%). The high burden of anemia in early infancy is likely attributable to rapid depletion of prenatal iron stores, increased iron requirements during rapid growth, and inadequate intake of iron-rich complementary foods after six months, as breast milk alone becomes insufficient to meet iron needs [40]. In contrast, the higher prevalence of undernutrition among children aged 13–36 months and 37–59 months likely reflects suboptimal complementary feeding practices, poor dietary diversity, recurrent infections, and the cumulative effects of prolonged nutritional inadequacies during early childhood, even as anemia prevalence declines with age due to dietary diversification [15,41]. This age-specific patterns of anemia and undernutrition underscore the need for differentiated nutritional strategies tailored to the distinct developmental and nutritional vulnerabilities of children across early life stages.

A systematic review reported the pooled prevalence rates of wasting-anemia and stunting-anemia in least-developed countries to be 5.4% and 19.5%, respectively [42]. These rates are higher than our findings from Nepal, which were 4.0% and 12.5%, respectively.

The high prevalence of coexistence of undernutrition and anemia in our study (16%) suggests that affected children are caught in an interlinked cycle of micronutrient deficiencies. Undernutrition impairs iron absorption and utilization, while anemia exacerbates undernutrition by reducing the body’s ability to effectively utilize nutrients.

The children from the richest wealth quintile had lower odds of having the coexistence of anemia and undernutrition compared to those from the poorest quintile. This wealth-related equity gap in undernutrition among children aged 6–59 months may be explained by the household economic status, which influences food insecurity and health care utilization when a child is ill [43–45].

Children from families in which mothers participated in household decision-making and mothers with secondary or higher education were less likely to experience the coexistence of anemia and undernutrition, as well as anemia alone, compared to children without these conditions. The health and nutrition of the child largely depend on the mother, who is typically the primary caretaker and responsible for awareness of the child’s health. Analyses of DHS data from Nepal and India suggest that women’s decision-making authority (maternal autonomy in household decision-making) improves children’s nutritional status and reduces child mortality, even after controlling for education and wealth [46].

## Strengths and limitations

This study has several strengths. First, it utilized complex survey analysis to account for survey design and non-response rates. Second, the findings are generalizable to children aged 6–59 months in Nepal and to children of the same age group in demographically and economically similar countries, as the study used a nationally representative dataset.

This study has several limitations. First, due to the missing data, we could not include some of the important dietary factors like minimum dietary diversity score and child’s birth weight. Second, because of the cross-sectional nature of the study, the directionality of associations cannot be established. Third, the results from this study may not be generalizable to populations outside Nepal with different demographic and economic status. Lastly, the wide confidence intervals observed in the associations between children’s nutritional status and anemia may suggest limited statistical power, likely due to small sample sizes within certain subgroups.

## Conclusion

The prevalence of undernutrition, anemia, and their coexistence remains high among children aged 6–59 months in Nepal. The coexistence of anemia and undernutrition was less likely among children from the highest wealth quintile, those whose mothers attained at least secondary level education, and those whose mothers participated in household decision-making. In contrast, coexistence was more likely among children whose mothers had poor maternal nutritional status. It is essential to implement holistic programs and targeted interventions to empower poor families, improve mothers’ education, strengthen women’s roles in household decision-making, and ultimately improve the children’s health.
